# Case report: Infantile pulmonary alveolar proteinosis associated with cytosolic isoleucyl-tRNA synthetase deficiency

**DOI:** 10.3389/fphar.2025.1487993

**Published:** 2025-01-30

**Authors:** Jie Wu, Yimu Fan, Feng Huo, Jie Deng, Quan Wang, Yuelin Shen

**Affiliations:** ^1^ Emergency Department, Beijing Children’s Hospital, Capital Medical University, National Center for Children’s Health, Beijing, China; ^2^ Neurology Department, Beijing Children’s Hospital, Capital Medical University, National Center for Children’s Health, Beijing, China; ^3^ Pediatric Intensive Care Unit, Beijing Children’s Hospital, Capital Medical University, National Center for Children’s Health, Beijing, China; ^4^ Respiratory Department II, National Clinical Research Center for Respiratory Diseases, Beijing Children’s Hospital, Capital Medical University, National Center for Children’s Health, Beijing, China; ^5^ Respiratory Department, Children’s Hospital Affiliated to Zhengzhou University, Henan Children’s hospital, Zhengzhou Children’s Hospital, Zhengzhou, China

**Keywords:** IARS1 gene, pulmonary alveolar proteinosis (PAP), infant, gene muatation, case repoort

## Abstract

Cytosolic isoleucyl-tRNA synthetase (IARS1) deficiency, an exceptionally rare autosomal recessive inherited disorder, is characterized by multiple system involvement, including growth retardation, intellectual developmental disorder, hypotonia, and hepatopathy. Pulmonary alveolar proteinosis (PAP) is a rare phenotype of IARS1 deficiency, having been reported in only two siblings from the same family. In this study, we present a case of IARS1 deficiency in a 5-month-old boy, who exhibited PAP as the initial and predominant manifestation. Additionally, whole-exome sequencing identified compound heterozygous variants in the *IARS1* gene (c.2428C>T/c.128T>C), both of which are novel observations.

## Introduction

Aminoacyl-tRNA synthetases (ARSs) constitute an evolutionarily ancient family of enzymes. Their primary function is to catalyze the esterification reaction that links a transfer RNA (tRNA) with its cognate amino acid, matching the anticodon triplet of the tRNA. Proper functioning of ARSs are essential for efficient and accurate protein synthesis (Fuchs et al., 2019). To date, over 60 human genetic diseases caused by pathogenetic variants in *ARS* genes have been documented (Wallen and Antonellis, 2013; Konovalova and Tyynismaa, 2013; Okamoto et al., 2022). Pathogenic variations leading to recessive ARS deficiencies typically occur within the catalytic or anticodon binding domains of ARS genes. Consequently, the prevailing hypothesis suggests that aminoacylation may be insufficient to meet translational demands in specific organs, particularly during periods of heightened demand, such as the first year of life and during infections. The *IARS1* gene encodes the cytosolic isoleucyl-tRNA synthetase (IARS1), which belongs to the class 1 family of ARSs. IARS1 deficiency, an extremely rare autosomal recessive inherited disorder, is characterized by multiple system involvement, including growth retardation, intellectual disability, muscular hypotonia, and infantile hepatopathy (GRIDHH, OMIM 617093). In this study, we reported a case of IARS1 deficiency in a 5-month-old Chinese boy who presented with a rare phenotype of pulmonary alveolar proteinosis (PAP) as the initial and predominant manifestation.

## Case report

A 5-month-old Chinese male infant presented with respiratory distress and growth retardation since birth. He is the firstborn child in the family, delivered vaginally at 37 weeks with a birth weight of 2,400 g. The parents are non-consanguineous and have no reported family history of genetic diseases. Following birth, he received nasal continuous positive airway pressure support for 10 days, which partially improved his respiratory distress but did not completely resolve it. At 2 months of age, he was admitted to the intensive care unit due to ongoing respiratory distress, hypoxemia (oxygen saturation on room air ranged from 75% to 90%), pneumonia and diarrhea. Although he responded to respiratory support and symptomatic treatment, he remained oxygen-dependent. By 5 months of age, his respiratory distress and hypoxemia had progressively worsened, necessitating referral to Beijing Children’s Hospital for further investigation and management.

Laboratory tests revealed a normal temperature of 36.2°C, tachycardia (heart rate of 180 beats per minute), and tachypnea (respiratory rate of 70 breaths per minute). His blood pressure was 87/50 mmHg, and oxygen saturation on room air was 86%. Notably, the patient presents with severe malnutrition, as evidenced by his low body measurements: a height of 59 cm (below the 3rd percentile) and a weight of 3.5 kg (also below the 3rd percentile). His physical examination reveals microcephaly, sparse hair, scant subcutaneous fat, poor head control, and an inability to turn over. Additionally, he exhibits cyanosis in his complexion and lips, reduced breath sounds on bilateral lung auscultation, decreased muscle tone, and muscle strength graded as III.

**FIGURE 1:**
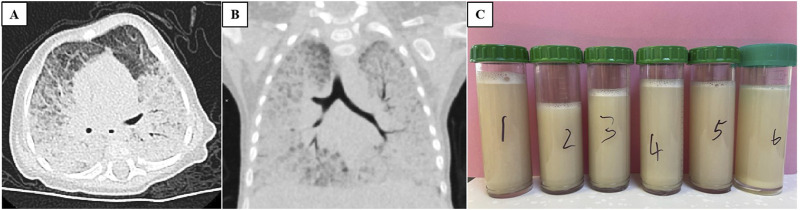
Chest CT scan (**(A)** transverse view and **(B)** coronal view) both showing widespread ground-glass opacities in both lungs, along with thickened interlobular septa. **(C)** Bronchoalveolar lavage fluid appeared milky white.

The patient exhibits leukocytosis (white blood cell count ranging from 19.37 to 22.01 × 10^9^/L), with lymphocytes being predominant (52%–68%). C-reactive protein and procalcitonin levels are within the normal range. Arterial blood gas analysis revealed a pH of 7.368, PaO_2_ of 58.5 mmHg, and PaCO_2_ of 47.3 mmHg during nasal cannula oxygen therapy (2 L/min). Biochemical analysis showed elevated alanine aminotransferase levels (67–115 U/L) and decreased albumin (24–29 g/L). Serum Krebs von den Lungen-6 was markedly increased (7470 U/mL; normal range 102–460 U/mL). Tuberculin skin test, interferon-γ release assay, HIV antibody testing, and metabolic screening were negative. Echocardiography revealed a patent foramen ovale. Abdominal ultrasound demonstrated a normal liver size with increased echogenicity in the hepatic parenchyma. Chest X-ray revealed diffuse lung infiltrate. Chest high-resolution computed tomography (HRCT) showed widespread ground-glass opacities in both lungs, along with thickened interlobular septa (Figures 1A, B). Bronchoalveolar lavage fluid (BALF) appeared milky white (Figure 1C), and periodic acid-Schiff (PAS) staining was positive. However, both culture and next-generation sequencing-based microbial identification were negative. These findings suggest PAP. To further clarify the etiology of infantile PAP, the patient and his parents underwent whole-exome sequencing (WES).

**FIGURE 2:**
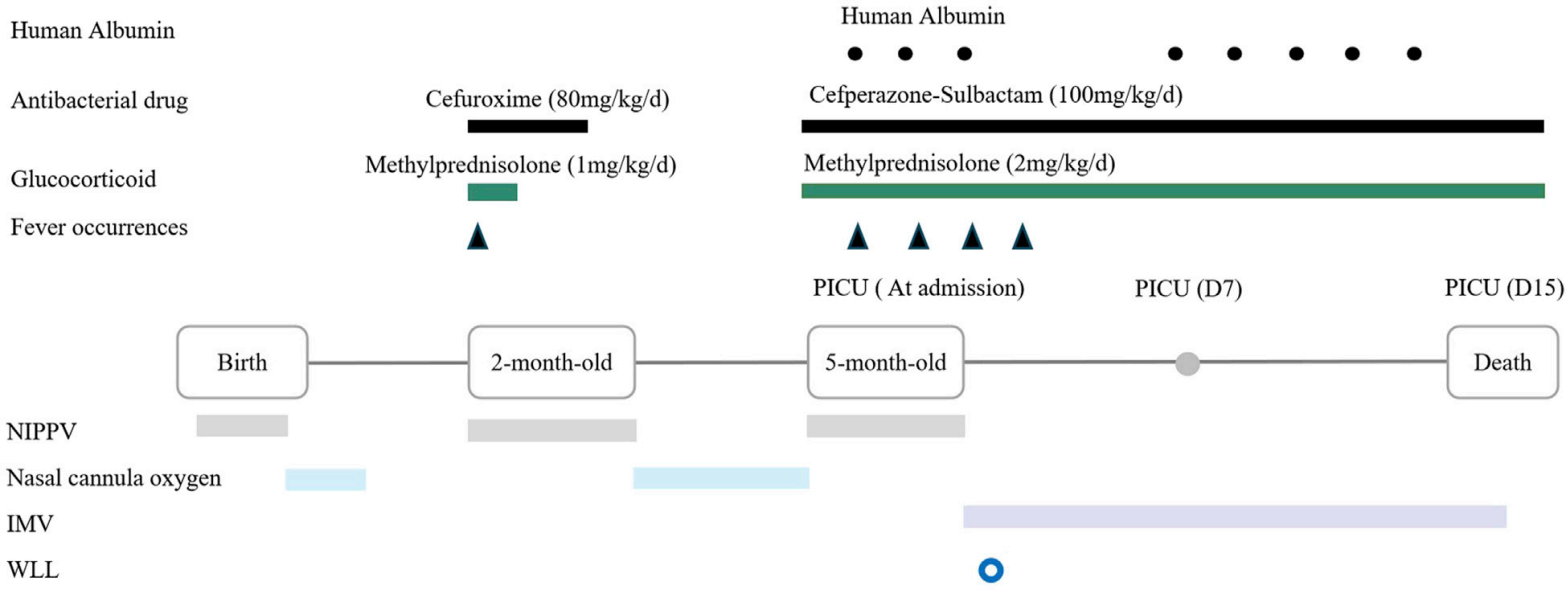
Timeline of medical history from birth to death in a 5-month-old boy with IARS1 deficiency. Abbreviation: NIPPV = Non invasive positive pressure ventilation; IMV = Intermittent mandatory ventilation; WLL = Whole lung lavage.

The patient received intensive care support. Upon admission, as nasal cannula oxygen could not improve hypoxemia, non invasive positive pressure ventilation was initiated. Two days later, the patient developed fever and worsening hypoxemia, prompting a switch to invasive mechanical ventilation. Intravenous cefperazone-sulbactam (100 mg/kg/d) and methylprednisolone (2 mg/kg/d) were administered, and albumin supplementation was provided intermittently for hypoproteinemia. Due to severe malnutrition, high-calorie formula milk was given, with a daily protein intake of approximately 2.6 g/kg. However, these treatments did not yield any improvement in symptoms. On the third day of admission, whole lung lavage (WLL) was performed. Following WLL therapy, there was only transient improvement in hypoxemia (oxygen saturation up to 90% at 40% FiO_2_). However, after 6 days, the patient’s respiratory distress continued to deteriorate, with oxygen saturation decreased to 84% at 80% FiO_2._ Due to severe hypoxemia, he could not tolerate a second WLL, ultimately leading to respiratory failure and death on the 15th day of hospitalization (Figure 2). The patient’s family declined an autopsy.

**FIGURE 3:**
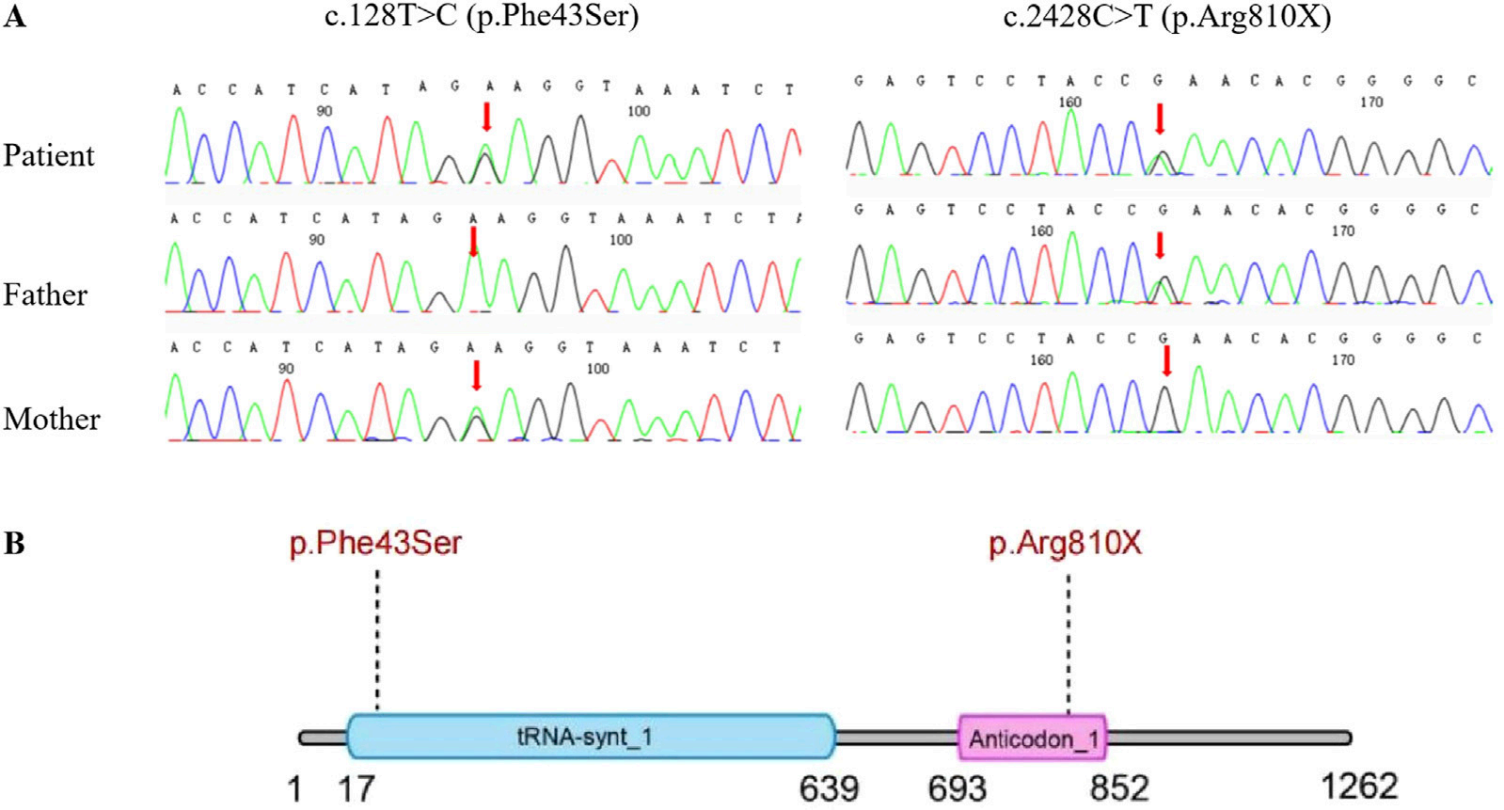
**(A)** Sanger DNA sequencing of the *IARS1* gene in a 5-month-old boy with IARS1 deficiency and the carrier status of his parents. **(B)** Schematic representation of the domain structure of the IARS protein, indicating the locations of the variants.

Two weeks after the patient’s death, WES results were finally available, identifiing compound heterozygous variants (c.2428C>T [p.Arg810X] and c.128T>C [p.Phe43Ser]) in the *IARS1* gene, inherited from the father and mother, respectively (Figure 3). Additionally, both of these variants are novel observations. These findings eventually confirmed the diagnosis of IARS1 deficiency.

## Discussion

In this study, the patient exhibited PAP as the initial and predominant manifestation, accompanied by multi-system involvements such as microcephaly, growth retardation, developmental delay, hypotonia, and infantile hepatopathy. WES identified compound heterozygous variants in the *IARS1* gene, which eventually confirmed the diagnosis of IARS1 deficiency. This condition was first reported by Kopajtich et al. (2016) and was named GRIDHH (Kopajtich et al., 2016). To date, only eleven cases (from ten families, including the current case) of IARS1 deficiency have been reported worldwide (Table 1) (Fuchs et al., 2019; Kopajtich et al., 2016; Orenstein et al., 2017; Smigiel et al., 2017; Fagbemi et al., 2020; Zou et al., 2022; Jiang et al., 2024).

**TABLE 1:** Cases of *IARS1* deficiency reported in the literature.

Case	Ref.	Age at Dx, y	Sex	Clinical phenotype					Genotype
				Digestive disorder	Neurological disorder	Respiratory disorder	Growth and development disorder	Others	
1	Kopajtich et al. (2016)	18.7	M	Hypoalbuminemia/Liver dysfunction	Spastic movement disorder/Muscular hypotonia/Microcephaly	Recurrent pneumonia	Growth retardation/Intellectual disability	Zinc deficiency/Esophagitis	c.1252C>T (p.Arg418X)/c.3521T>A (p.lle1174Asn)
2	Kopajtich et al. (2016)	19.0	F	Liver dysfunction	Epilepsy	None	Growth retardation/Intellectual disability	Zinc deficiency/Diabetes mellitus/Sensoneurinal hearing loss	c.760C>T (p.Arg254X)/c.1310C>T (p.Pro437Leu)
3	Kopajtich et al. (2016)	3.0	M	Hypoalbuminemia/Liver dysfunction	Muscular hypotonia/Microcephaly	Recurrent pneumonia	Growth retardation/Development delay	Zinc deficiency	c.1109T>G (p.Val370Gly)/c.2974A>G (p.Asn992Asp)
4	Orenstein et al. (2017)	4.0	M	Hypoalbuminemia/Liver dysfunction	Microcephaly	None	Growth retardation/Development delay	Hydronephrosis/Joint hyperlaxity/Hypoglycemia	c.2215C>T (p.Arg739Cys)/c.1667T>C (p.Phe556Ser)
5	Smigiel et al. (2017)	7.0	M	Liver dysfunction	Muscular hypotonia/Microcephaly	Recurrent pneumonia	Growth retardation/Intellectual disability	Zinc deficiency/Clubbing	c.2011del (p.Gln671ArgfsX6)/c.206C>T (p.Thr69lle)
6–1	Fuchs et al. (2019)	0.3	F	Hypoalbuminemia/Liver dysfunction	Microcephaly	PAP	Growth retardation/Developmental delay	None	c.1305G>C (p.Trp435Cys)/c.3377dup (p.Asn1126LysfsX9)
6–2	Fuchs et al. (2019)	5.0	M	Hypoalbuminemia/Liver dysfunction	Muscular hypotonia/Microcephaly	Recurrent pneumonia/PAP	Growth retardation/Intellectual disability	None	c.1305G>C (p.Trp435Cys)/c.3377dup (p.Asn1126LysfsX9)
7	Fagbemi et al. (2020)	9.0	M	Inflammatory bowel disease/Liver dysfunction	Microcephaly	None	None	None	c.290A>G (p.Asp97Gly)/c.290A>G (p.Asp97Gly)
8	Zou et al. (2022)	1.6	F	Hypoalbuminemia/Liver dysfunction/Diarrhea	Muscular hypotonia/Microcephaly	Recurrent pneumonia	Growth retardation/Development delay	Vitamin D deficiency	c.701T>C (p.Leu234Pro)/c.1555C>T (p.Arg519Cys)
9	Jiang et al. (2024)	1.4	F	Hypoalbuminemia/Liver dysfunction	Epilepsy/Muscular hypotonia/Microcephaly	None	Growth retardation/Development delay	None	c.120-1G>A/c.2164C>A (p.Arg722Ser)
10	Current report	0.4	M	Hypoalbuminemia/Liver dysfunction/Diarrhea	Muscular hypotonia/Microcephaly	PAP	Growth retardation/Developmental delay	None	c.2428C>T (p.Arg810X)/c.128T>C (p.Phe43Ser)

Abbreviations: Dx, Diagnosis; PAP, Pulmonary alveolar proteinosis; Ref, Reference.

Patients with IARS1 deficiency exhibit significant phenotypic heterogeneity and diversity, ranging from mild clinical manifestations to rapidly progressive involvement of multiple organ systems. The phenotype can vary considerably among different individuals within the same family as well as between distinct families. Among the reported patients, there were 7 males (63.6%) and 4 females (36.3%). The most common phenotype observed was liver disease (11 patients, 100%), along with growth and developmental disorders (10 patients, 90.9%). Neurological manifestations varied and included features such as microcephaly (10 patients, 90.9%), muscular hypotonia (7 patients, 63.7%), and epilepsy (2 patients, 18.2%). Within the respiratory system, recurrent pneumonia was the predominant phenotype (5 patients, 45.5%). Notably, three patients (27.3%) exhibited a rare PAP. Apart from the current case, the other two cases were siblings from the same family, reported by Fuchs et al. (2019) (Case 6-1 and Case 6-2 in Table 1). They both experienced respiratory distress and oxygen dependence since infancy. Their chest CT scans revealed diffuse interstitial changes, and the final pathological diagnosis confirmed PAP.

PAP is a rare disease characterized by the abnormal surfactant accumulation within the alveoli, resulting in progressive respiratory insufficiency (Jouneau et al., 2020). The childhood PAP classification scheme generally includes the following categories (Bush and Pabary, 2020): Disorders related to surfactant protein metabolism (involving *SFTPB*, *STFPC*, *ABCA3* and *NKX2-1* mutations); GM-CSF receptor gene mutations (including *CSF2RA*/*CSF2RB* mutations); other genetic disorders (including *ARS*, *STING*, *COPA* and *GATA2* mutations); metabolic disease (such as lysinuric protein intolerance, Niemann-Pick disease); secondary PAP (such as immunodeficiency, connective tissue disease), and autoimmune PAP (caused by IgG autoantibodies to GM-CSF) (Table 2). The classification of PAP in adult is not always applicable to children, in whom the autoimmune form is exceptionally rare, and genetic causes predominate. In this case, the patient presented with respiratory distress and hypoxemia since birth. Chest HRCT scans revealed the classic “crazy-paving patten” sign associated with PAP. BALF appeared milky, and positive PAS staining confirmed the diagnosis of PAP. Additionally, the patient exhibited multi-system symptoms, including microcephaly, growth retardation, developmental delay, hypotonia, and infantile hepatopathy, with an early disease onset, which suggests an association with a genetic disorder. We ruled out autoimmune PAP due to negative anti-GM-CSF antibodies. Whole exome sequencing excluded defects in surfactant metabolism pathways, GM-CSF receptor gene mutations, as well as primary immunodeficiencies or inherited metabolic disorders. Therefore, the most likely cause of PAP in this patient appears to be *IARS1* deficiency. The diagnostic challenges in this infant patient stem from nonspecific clinical symptoms. Fever and respiratory distress are common to other respiratory diseases, leading to potential misdiagnosis. Additionally, infantile PAP is a rare disease, and physicians often lack recognition and experience with it, which can cause diagnostic delays. Furthermore, the etiology of infantile PAP is complex and frequently requires WES, a time-consuming and costly process. Interestingly, there have been reports of PAP associated with mutations in other *ARS* genes (such as *MARS1* and *FARS1*) (Hadchouel et al., 2015; Schuch et al., 2021). The association between ARS deficiency and PAP is not yet fully understood. Recent study suggest that MRS deficiency disrupts surfactant composition or homeostasis, potentially contributing to PAP (Hadchouel et al., 2022). Further research is needed to unravel the precise mechanisms underlying PAP in the context of *IARS1* deficiency and other *ARS* mutations.

**TABLE 2:** Proposed classification of pediatric PAP.

Category of disease	Exemplar conditions
Disorders related to surfactant protein metabolism	*SFTPB, STFPC*, *ABCA3* and *NKX2-1* mutations
GM-CSF receptor gene mutations	*CSF2RA*/*CSF2RB* mutations
Other genetic disorders	*ARS*
Metabolic disease	Lysinuric protein intolerance, Niemann-Pick disease
Secondary PAP	Immunodeficiency, Connective tissue disease
Autoimmune PAP (mainly in adults)	IgG autoantibodies to GM-CSF

The *IARS1* gene, located at 9q22.31, comprises 34 exons. Among the 11 patients with IARS1 deficiency, nine exhibited a compound heterozygous genotype, while only one had a homozygous genotype. A total of 17 mutations had been identified according to HGMD, including 14 missense/nonsense mutations, 1 splice site mutation, 1 small deletion, and 1 small duplication. Notably, we discovered two novel variants in this infant. The paternal variant, c.2428C>T (p.Arg810X), represents a nonsense variant resulting in the 810th amino acid changing from arginine to a stop codon. This alteration likely impacts protein function and is highly suggestive of pathogenicity. The maternal variant, c.128T>C (p.Phe43Ser), is a missense variant predicted to be deleterious by four bioinformatics prediction tools: Polyphen2, SIFT, Mutation Taster, and REVEL. These two novel variants expand the genetic spectrum associated with *IARS1*. Currently, no established genotype-phenotype correlation has been established.

There is no definitive cure for *IARS1* deficiency, and consensus on treatment remains elusive. Current management strategies primarily focus on supportive measures, including liver protection, nutritional support, supplementation of micronutrients and vitamins, and prevention of respiratory tract infections (Fuchs et al., 2019; Kopajtich et al., 2016). For patients with coexisting PAP, WLL is considered an effective treatment approach. This procedure involves clearing the accumulation of alveolar lipoproteins, improving oxygenation, and slowing disease progression. In this infant case, the WLL transiently improved hypoxemia, but the effect was short-lived, potentially due to concurrent severe infection. Additionally, the safety of WLL in infant cases remains inconclusive. Six days after the WLL, the patient’s respiratory distress continued to deteriorate. Due to the worsening hypoxemia and safety concerns, a second WLL was not performed. According to the literature, Fuchs et al. (2019) did not specify whether Case 6-1/6-2 underwent WLL. Case 6-1 succumbed at 4 months of age, while Case 6-2 experienced recurrent pneumonia and respiratory distress, necessitating 5 admissions to the pediatric intensive care unit within 2 years (Fuchs et al., 2019; Kok et al., 2021). As a result, Case 6-2 received supplementation with high-dose L-isoleucine (35–70 mg/kg/day in three doses). Notably, this approach led to improvements in infection frequency, chest imaging findings, oxygen dependence, growth and development. Additionally, Hadchouel et al. (2022) reported 4 cases of PAP due to MARS1 deficiency, where methionine supplementation significantly ameliorated multi-system impairments. Lenz et al. (2020) also described a case of PAP associated with MARS1 deficiency, where the patient experienced respiratory distress unresponsive to invasive high-frequency oscillatory ventilation, nitric oxide, and corticosteroids. Subsequent oral administration of methionine, starting at an initial dose of 50 mg/kg and gradually increasing to 125 mg/kg, along with a daily protein intake of 2–3 g/kg, resulted in clinical improvement. The patient no longer required ventilatory and oxygen support, and imaging showed improvement in interstitial lung disease. These findings suggest that if patients with ARS deficiencies retain some residual enzyme activity, supplementation with the corresponding amino acid or a high-protein diet may be beneficial. However, in our case, it was observed that a daily protein intake of 2.6 g/kg during hospitalization did not improve the outcome. Based on the outcome reported in Case 6-2, we hypothesize that supplementation with isoleucine may be more crucial for our patient. Future research should explore the potential benefits of isoleucine supplementation in patients with IARS deficiency, as well as determine the optimal timing and dosage for such supplementation.

The limitation of the present study is that, due to the lengthy duration of genetic testing and the patient’s rapid deterioration and death shortly after admission, the WES results were only available 2 weeks after the patient’s death. Therefore, during hospitalization, we did not have a definitive diagnosis and did not have the opportunity to intervene with isoleucine supplementation or conduct long-term follow-up. However, we hope that this case report of the rare disease will draw attention to PAP due to IARS1 deficiency.

In conclusion, this is a rare case of IARS1 deficiency presenting with infantile PAP as the initial and predominant manifestation, with a poor prognosis. There is currently no definitive cure for IARS1 deficiency. WLL can transiently improve hypoxemia, but the duration of improvement and safety in infant cases remains inconclusive. Future research should explore the potential benefits of isoleucine supplementation in patients with IARS deficiency.

## Data Availability

The original contributions presented in the study are included in the article/supplementary material, further inquiries can be directed to the corresponding authors.
